# Population-Level Interventions Targeting Risk Factors for Hypertension and Diabetes in Rwanda: A Situational Analysis

**DOI:** 10.3389/fpubh.2022.882033

**Published:** 2022-07-01

**Authors:** Jean Pierre Nganabashaka, Seleman Ntawuyirushintege, Jean Berchmans Niyibizi, Ghislaine Umwali, Charlotte M. Bavuma, Jean Claude Byiringiro, Stephen Rulisa, Jacob Burns, Eva Rehfuess, Taryn Young, David K. Tumusiime

**Affiliations:** ^1^College of Medicine and Health Sciences, University of Rwanda, Kigali, Rwanda; ^2^Institute for Medical Information Processing, Biometry, and Epidemiology, Faculty of Medicine, Ludwig Maximilian University of Munich, Munich, Germany; ^3^Pettenkofer School of Public Health, Munich, Germany; ^4^Centre for Evidence-Based Health Care, Department of Global Health, Stellenbosch University, Cape Town, South Africa

**Keywords:** population-level interventions, physical inactivity, unhealthy diet, alcohol use, tobacco use, diabetes, hypertension, Rwanda

## Abstract

**Background:**

Eighty percent (80%) of global Non-Communicable Diseases attributed deaths occur in low- and middle-income countries (LMIC) with hypertension and diabetes being key contributors. The overall prevalence of hypertension was 15.3% the national prevalence of diabetes in rural and urban was 7.5 and 9.7%, respectively among 15–64 years. Hypertension represents a leading cause of death (43%) among hospitalized patients at the University teaching hospital of Kigali. This study aimed to identify ongoing population-level interventions targeting risk factors for diabetes and hypertension and to explore perceived barriers and facilitators for their implementation in Rwanda.

**Methods:**

This situational analysis comprised a desk review, key informant interviews, and stakeholders' consultation. Ongoing population-level interventions were identified through searches of government websites, complemented by one-on-one consultations with 60 individuals nominated by their respective organizations involved with prevention efforts. Semi-structured interviews with purposively selected key informants sought to identify perceived barriers and facilitators for the implementation of population-level interventions. A consultative workshop with stakeholders was organized to validate and consolidate the findings.

**Results:**

We identified a range of policies in the areas of food and nutrition, physical activity promotion, and tobacco control. Supporting program and environment interventions were mainly awareness campaigns to improve knowledge, attitudes, and practices toward healthy eating, physical activity, and alcohol and tobacco use reduction, healthy food production, physical activity infrastructure, smoke-free areas, limits on tobacco production and bans on non-standardized alcohol production. Perceived barriers included limited stakeholder involvement, misbeliefs about ongoing interventions, insufficient funding, inconsistency in intervention implementation, weak policy enforcement, and conflicts between commercial and public health interests. Perceived facilitators were strengthened multi-sectoral collaboration and involvement in ongoing interventions, enhanced community awareness of ongoing interventions, special attention paid to the elderly, and increased funds for population-level interventions and policy enforcement.

**Conclusion:**

There are many ongoing population-level interventions in Rwanda targeting risk factors for diabetes and hypertension. Identified gaps, perceived barriers, and facilitators provide a useful starting point for strengthening efforts to address the significant burden of disease attributable to diabetes and hypertension.

## Introduction

Non-communicable diseases (NCDs) claim about 41 million deaths each year, equivalent to 71% of global deaths (1), of which 80% is in low- and middle-income countries (LMIC).(2, 3). Both hypertension and diabetes are major contributors to NCD deaths (4). Hypertension is a key risk factor for cardiovascular diseases (CVDs) (5). Globally, an estimated 1.13 billion people have hypertension, two-thirds are from low-middle-income countries (LMICs) (6). More than 1.6 million deaths in 2015 were due to diabetes, while 422 million adults live with diabetes (5).

People with diabetes are at high risk of CVDs (7, 8). The number of people living with diabetes in Sub-Saharan Africa (SSA) was estimated at 12.1 million people in 2010; this number is expected to rise to 23.9 million by 2030 (9, 10) and to 47.1 million by 2045 (11), with the onset at a younger age in LMICs (12, 13). In Rwanda, the national prevalence of diabetes in rural and urban was 7.5 and 9.7%, respectively among 15–64 years (14) whereas the overall prevalence of hypertension was 15.3% (16.4% for males and 14.4% for females) (15). Hypertension was a leading cause of death (43%) among hospitalized patients at the University teaching hospital of Kigali in Rwanda (16). The latest published NCDs risk factors survey revealed that 19% of men and 7% of women were tobacco smokers, 30% of men and 17% of women had binged on alcohol in the past 30 days and 99% of participants consumed <5 servings of fruit and/or vegetables per day (17). The majority of NCDs can be prevented through various preventive measures including policies and various interventions (18). The sixty-sixth World Health Assembly (WHA) held in 2013 called for a 25% reduction in NCD deaths by 2025 (19). A study done in China, India, and Mexico showed that community-based interventions for health are scalable to the population-level, affordable, and effective in controlling risk factors for NCDs in LMICs (20). Beyond the WHA target, NCDs Synergies network and the Rwanda Ministry of Health (MoH) have set the target to reduce 80% of NCD-related deaths among people under 40 years of age by 2020, through various population-level interventions and integrated health service delivery platforms (21). This ambition was believed to be achieved through leveraging the existing investments. Rwanda went ahead to implement some of the population-level interventions. Population-level interventions are made up of supporting environment (activities influencing the creation of environment in which healthy choices are the easier option for the population; example: playgrounds for physical activity), supporting policies (fiscal, legislative, and regulatory measures that foster and promote healthy choices; example: tobacco control policies) and programs (activities implemented at all levels to promote healthy choices of the community; example: salt intake reduction awareness campaign) (22). It is not exactly known which and to what extent population-level interventions are being implemented in Rwanda. This study aimed to provide a comprehensive overview of the currently implemented population-level interventions targeting risk factors for diabetes and hypertension, as well as to explore perceived barriers and facilitators affecting implementation using multiple approaches.

## Methods

### Study Design, Sampling and Data Collection

This situational analysis was conducted in parallel across three African countries (Rwanda, Malawi, and South Africa) using a standardized but locally adapted protocol. In Rwanda, which is described in this study, the situational analysis was conducted from February to September 2019. It comprised a desk review, semi-structured interviews with purposively selected key informants, and a one-day consultative workshop.

The desk review consisted of two phases: an online search of policy documents and consultations with selected stakeholders. In the first phase, we searched the government website (https://www.gov.rw/) to obtain relevant policy documents from various ministries and agencies involved in population-level interventions targeting risk factors for diabetes and hypertension. Additional documents were requested directly from key stakeholders, specifically from Rwanda MoH and the Rwanda Biomedical Center (RBC). Retrieved published and gray literature were searched using terms such as diabetes mellitus or hypertension, population, prevention, and Rwanda plus a risk factor of interest. A document was eligible for in-depth review if it contained at least one of the information regarding the design, implementation or evaluation of population-level interventions to address NCD risk factors in Rwanda. An Excel spreadsheet was designed to extract relevant information on what are the implemented population-level interventions and the extent of their implementation (target population, coverage and the evaluation status) from all eligible documents. In the second phase of the desk review, we used information from the first phase to map several stakeholders involved in implementation of population level interventions. The mapping for stakeholders started with purposive reaching out to relevant personnel from the Ministries of Health and other governmental or non-governmental agencies already known to be actively involved in implementation of population level interventions and then using snow-balling method, 40 various key stakeholders (governmental or non-governmental) were mapped. Forty mapped institutions and organizations through a written letter, they were introduced to the current study and requested to nominate individuals who are knowledgeable in implementation of population level interventions of interest to talk to in a one-on-one (face-to-face) consultation, these were mainly people who are current working in NCDs unit in health sector, NCDs focal person or work closely with NCD technical working group for non-health sector. As a result, 60 individuals across different managerial and technical levels of these organizations were nominated and voluntary agreed to participate in the study. In some institutions, more than one person agreed to participate in our study. After obtaining their signed consent to participate in the study, a trained data collector used a designed data extraction sheet to collect their insights on ongoing population-level interventions in Rwanda. The information identified in this manner complemented data from the online search to provide a comprehensive overview of population-level interventions targeting risk factors for diabetes and hypertension implemented in Rwanda.

Semi-structured key informant interviews were conducted with a sub-set of key informants from across concerned institutions. Active involvement with ongoing population-level interventions as well as the diversity of the interviewees (i.e., policymakers, implementing agencies, and non-governmental organizations) and managerial as well as technical knowledge were considered in selecting key informants. We targeted NCDs focal people, heads of NCDs unit and members of national NCD technical working group because they fulfilled all these above mentioned criteria. Ten participants were chosen for pragmatic reasons for key informants interview. We used a structured interview guide consisting of open-ended questions designed and pilot- tested for this study. Participants were asked of their view on what was hindering (perceived barriers) and facilitates (perceived facilitators) the implementation of population-level interventions. Interviews with participants were undertaken by trained interviewers (DJ, UI, GJB, NJ, and BC), took place at the participants' workplace and lasted for 20 to 30 min. Both recording and note taking were done to accurately capture the contents of the interviews. Following a preliminary analysis of the data, a one-day consultative workshop to validate and consolidate findings was held with at least one participant representing each of the institutions that participated in this study.

### Data Analysis

Desk review data (website searches and one-on-one consultations) and qualitative data (key informant interviews) were analyzed separately because they answered different objectives. For desk review, both data from website searches and one-on-one consultations were compiled in a spreadsheet. Two researchers (JPN&DKT) independently classified interventions as policies, programs, and supporting environment interventions per each of the four main NCD risk factors (unhealthy diet, physical inactivity, tobacco use, and harmful use of alcohol). They (researchers) compared their findings and resolved any disagreement through open discussion. This classification was also guided by World Health Organization (WHO) best buys recommended interventions (23), where for each of the recommended interventions per each of the main NCDs risk factors were considered. We also assessed the progress of WHO recommended best buys Interventions in Rwanda. The interventions were graded fully implemented where they are written in the documents and have related programs and supporting environment interventions. They are graded inadequate if they are written in the documents but we lacked its related programs and supporting environment interventions.

For qualitative data (semi-structured key informant interview data), thematic analysis was performed using Atlas ti-7 (24). To get familiar with data, two researchers (JPN&DT) independently read both the interview transcripts and field notes. Using inductive approach, they (researchers) coded two first transcripts together to agree on coding framework. Then, the remaining transcripts were coded independently. Emerged codes and themes were discussed between the two researchers seeking to achieve consensus; a third researcher (JBN) was involved in this discussion and helped with conflict resolution. All authors reviewed and commented on the outputs of the analysis.

### Ethical Considerations

All methods were performed in accordance with the ethical standards of the national research ethics committee and with the 1964 Helsinki declaration and its later amendments. The Rwanda National Ethics Committee approved this study (approval No.0025/RNEC/2019). Written consent was obtained from all participants, participation was voluntary and participants' confidentiality was assured at all steps of the study.

## Results

Generally, information from the desk review, the consultations, the semi-structured key informant interviews and the consultative workshop, were informative and complementary in providing insights regarding the aims of the study. The online search retrieved 178 documents of which, 18 documents (policies, laws, strategic plans, and Ministerial orders/instructions) describing population-level interventions targeting NCD risk factors were included. Sixty participants were visited at the workplace as part of the stakeholders' consultations (Table 1, left column). They provided information on programs and other population- level interventions beyond the identified policies from online desk review. Ten key informants were interviewed (Table 1, right column) and shared their perceptions regarding barriers and facilitators for the implementation of population-level interventions targeting NCD risk factors.

**Table 1:** Study participants in one-on-one consultations and key informants interviews.

**Participant Position**	**Number of participants**	
	Consultations	Key informants interviews
Division manager	10	2
Managing director	3	1
NGO director	4	1
Program manager	10	1
District directors of the health unit	12	2
NCD alliance member	21	3
**Total**	60	10

### Population-Level Interventions Targeting Risk Factors for Diabetes and Hypertension in Rwanda

**Figure 1:**
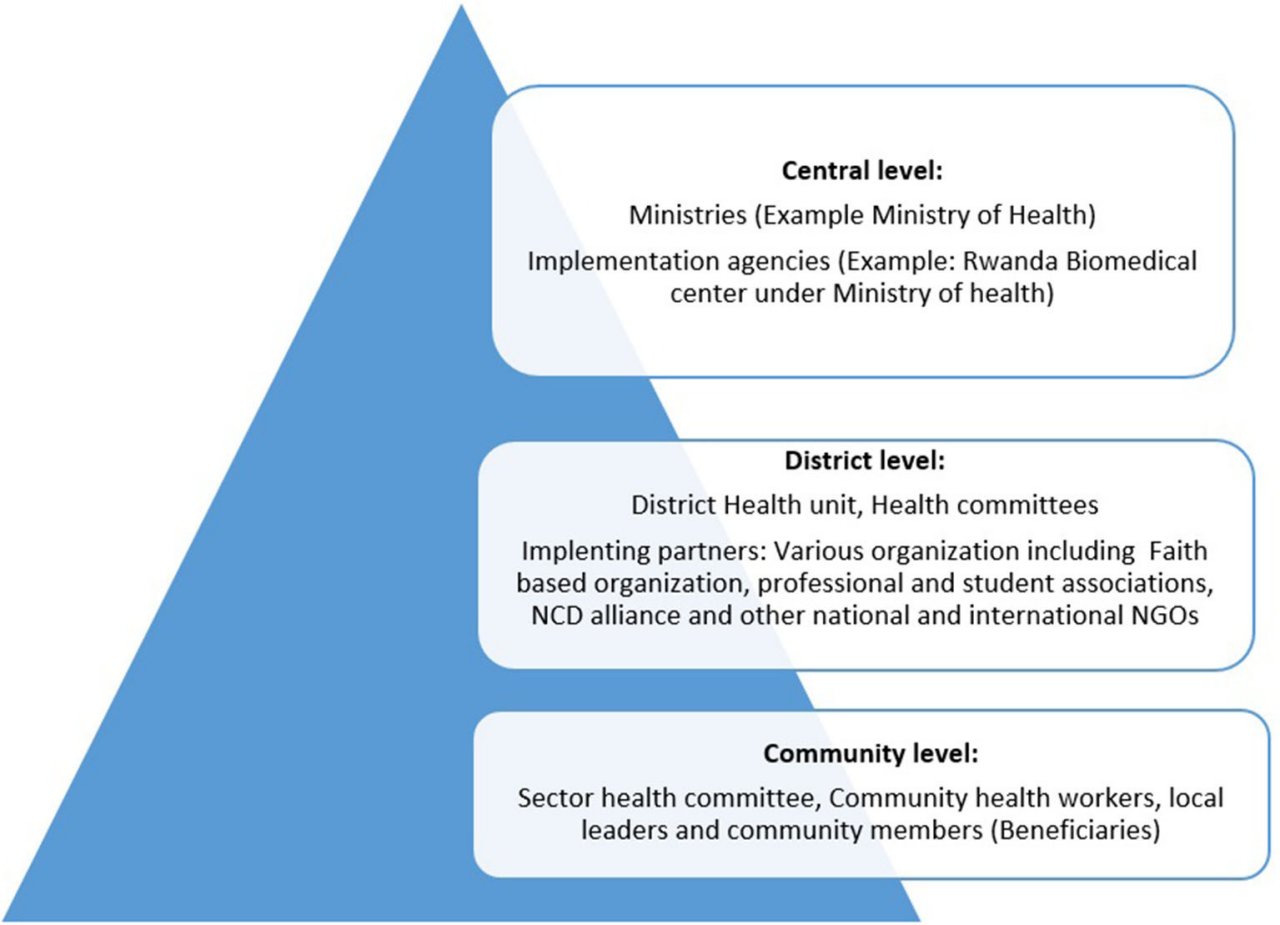
Flowchart of population-level interventions implementation in Rwanda.

Online searches and stakeholders' consultations identified population-level interventions implemented in Rwanda, comprising policies, laws, ministerial orders, notices and instructions, programs, and supporting environment interventions. Rwandan health system consists of three level namely central level which consists of ministries and affiliated implementing agencies, district level is the point of implementation and it consists of district health unit, health committee and various partners including national and international governmental organization (Figure 1). Tis flowchart is not only applicable to health sector but others too. Policies are developed at central level, implementing agencies breakdown policies into actions (program and supporting environment interventions) and the implementation is done at district by district itself or in partnership with various partners over guidance and coordination of ministry affiliated implementing agencies. Community level consisted of sector leaders and health committee at the health center who serve as a liaison to the community members (beneficiaries). The policies, laws, ministerial orders, notices and instructions target most of the population categories countrywide. These are implemented through designing of specific programs and supporting environment interventions. Programs and supporting environment interventions are sometimes specific to a given category of people and may cover a sub-set of the country depending on the priority and the availability of required resources. Desk review only identified documents describing the implementation phase of the interventions, but we did not identify any documents describing their evaluation. Depending on the risk factor considered, the concerned ministries and its partners established and coordinated the implementation of the respective interventions; for example, interventions targeting physical inactivity were established by the Ministry of sports (Table 2). The NCD Policy (25) and its Strategic Plan (26) (July 2014–June 2019), as well as the Fourth Health Sector Strategic Plan (27) of 2018–2024, provide overall guidance and strategies to tackle risk factors for diabetes, hypertension, and NCDs in general. The NCD policy is the main guiding document for most of the population-level interventions and defines the respective roles of each of the concerned stakeholders.

**Table 2:** Population—level policy interventions targeting risk factors for hypertension and diabetes being implemented in Rwanda.

**Targeted risk factors**	**Policy**	**What it covers**	**When it was established**	**Coordination leader**	**Target population**	**Coverage**	**Evaluation status**
Cross-cutting policy interventions targeting all 4 risk factors unhealthy diet, physical inactivity, tobacco use and harmful use of alcohol	Non-Communicable Disease (NCD) Policy	This Policy provides guidelines and strategies to target all NCDs risks factors.	2015	Ministry of health and Rwanda Biomedical Center (RBC)	All age categories	Countrywide	Not evaluated
	Rwanda NCDs national strategic plan		2014–2019		All age categories	Countrywide	Not evaluated
	4^th^ Health Sector strategic plan (HSSP IV)	HSSP IV is the guiding document that focuses on several interventions against various NCDs risk factors such as reinforcement of laws on tobacco and alcohol, promotion of physical activity and healthy diet	2018–2024	Ministry of health	All age categories	Countrywide	Not evaluated
	National school health policy	The National school health policy targets pupils from pre-primary to secondary schools to improve their learning outcomes. It guides various interventions targeting unhealthy, such as nutrition education, and physical activity promotion as well as prevent alcohol, tobacco and drug abuse prevention interventions.	2014	Ministry of education	Schooling children	Countrywide	Not evaluated
Policy interventions targeting unhealthy diet	National Food and Nutrition policy (NFNP)	This policy focuses on providing seeds for vegetable and fruits as well as the planting guidance. Provides a framework for food in emergencies and supports schools' feeding programs.	2013–2018	Social cluster ministries led by Ministry of health, Ministry of agriculture and animal resources, Ministry of education, Ministry of gender and family promotion and Ministry of local government	All age categories	Countrywide	Not evaluated
	National Early Childhood Development (NECD) policy	Focuses on combating malnutrition through different initiatives such as a program providing a cow per family, provision of fortified food to some households, and promoting fruit and vegetable farming at the community level through kitchen and village gardens.	2016	Ministry of gender and family promotion	Children from 0 to 6 years	Countrywide	Not evaluated
	National agriculture policy	This policy provides guidance on different nutritional awareness, education programs and availing bio-fortified food, promotion of kitchen gardens for vegetables and fruits, distribution of livestock in needy households.	2018	Ministry of agriculture and animal resources	All age categories	Countrywide	Not evaluated
Policy interventions targeting physical inactivity	Ministerial order N°02/Mifotra/15 of 09/06/2015	To establish mandatory 3–5 p.m. weekly reserved for physical activity in public service.	2015	Ministry of Labor and Public Services and the Ministry of Sports	Public servant	Countrywide	Not evaluated
	Prime Minister's Instructions no.001/03.003/03/2012 of 03/03/2012		2012		Public servant	Countrywide	Not evaluated
	Sport development policy	Provides guidelines for various interventions targeting physical inactivity; this includes the initiation of different physical activities across the different population categories, ensures required infrastructures and resources	2012	Ministry of Sports	All age categories	Countrywide	Not evaluated
Policy interventions targeting tobacco use	Ministerial order N°20/34 of 09/06/2015 determining the content and design of the warning put on the package of tobacco and tobacco products	This order determines the contents and design of the health warning messages on tobacco and related product packaging.	2015	Ministry of health	All age categories	Countrywide	Not evaluated
	Ministerial public notice banning Shisha ban	This notice warns the public against the use, advertisement and importation of water pipe tobacco known as shisha effective from 15/12/2017.	2017	Ministry of health	All age categories	Countrywide	Not evaluated
	Law governing narcotic drugs, psychotropic substances and precursors in Rwanda N°03/2012 du 15/02/2012	This law regulates the production, processing, distribution and use of narcotic drugs, psychotropic substances and precursors in Rwanda.	2012	Ministry of Justice	All age categories	Countrywide	Not evaluated
	Instructions of the minister of health N° 20/10 of 10/05/2005 relating to the protection of non-smokers and the environment against the effect tobacco use	These instructions intends to protect both non-smokers and the environment against the hazardous effects of public smoking. Prohibit public smoking and advises pregnant and breastfeeding women against smoking. It ensures the tobacco waste products and residuals proper disposal to avoid environmental destruction.	2005	Ministry of health	All age categories	Countrywide	Not evaluated
	Ministerial Order determining the characteristics of smoking areas and the content included in the notice displayed in the smoking area N° 20/33 du 09/06/2015	This order defines the design of smoking areas and the protection of non-smokers and obliges the establishment of places reserved for smoking with clear warnings against public smoking.	2015	Ministry of health	All age categories	Countrywide	Not evaluated
	Law relating to the control of tobacco N°08/2013 of 01/03/2013	It intends toestablish smoking free environments and prohibits the illegal trade and production of tobacco by certification of tobacco to be legally used in Rwanda.	2013	Ministry of Justice	All age categories	Countrywide	Not evaluated
Policy interventions targeting harmful use of alcohol	No specific policy available	-	-	-	-	-	-

The National School Health Policy (28) targets students in pre-primary, primary, and secondary schools with respect to different health problems diseases, including NCDs. These four policies cross-cut across multiple risk factors, including unhealthy diets, physical inactivity, tobacco use, and harmful use of alcohol. Interventions specific to each of these four major NCD risk factors are presented in the following paragraphs. Table 2 below summarizes the cross-cutting interventions as well as the interventions targeting specific NCD risk factors.

### Interventions Targeting Unhealthy Diet

We identified three policies targeting unhealthy diet through the online searches, namely the National Early Child Development Policy, the National Agriculture Policy and the National Food and Nutrition Policy. The National Early Child Development Policy aims to reduce malnutrition and stunting among below 6 years old children. The National Agriculture Policy addresses food and nutrition security in the community through education and awareness programs focusing on food production, whereas the National Food and Nutrition Policy focuses on providing strategies to improve knowledge and capacity of households for food and nutrition security in food insecure areas. Through the consultations, we identified various community-based activities aiming to enhance knowledge and skills of the population related to the preparation and consumption of a healthy diet. These include, for example, media dialogues with nutritionists and health professionals, especially during nutrition week. Healthy diet education was integrated into the existing community programs, such as the monthly community work usually known in the local language as “Umuganda.” The Baho Neza “live healthy” program is an integrated campaign aiming to ensure healthy and happy families; as part of this program, families are taught to prepare healthy diets. A healthy diet is part of the curriculum in primary and secondary schools. Training of nutritionists and other health care providers enables them to educate the population about a healthy diet at both health facilities and the community level.

### Interventions Targeting Physical Inactivity

We identified three policies targeting physical inactivity. The Prime Minister's Instructions No 001/03.0/03/03/2012 of 03/03/2012 (29) and the Ministerial order No 02/mifotra/15 of 09/06/2015 (30) enforce the participation of civil servants in physical activity by determining time and facilitation for physical activity. The Sport development policy (31) promotes sports talent detection, school, and sports among the general population (Table 2).

Through the stakeholder consultations, we identified various campaigns for community participation in physical activity. Identified activities include the bimonthly physical activity campaign known as “car-free day,” which was initiated by the city of Kigali in 2016 and is currently being extended to most other cities and towns in the country. During this campaign, cars and motorbikes are stopped from using main roads in the city/town from 7 a.m. to 10 a.m. to allow people to exercise freely at the designated grounds with guidance by experienced facilitators. Sports competitions between public and private institutions such as schools, the army, the police, ministries, and government administrative entities are also common. For example, the Umurenge Kagame cup is a sport competition (mainly football) financed by the President of the Republic of Rwanda His Excellency Paul Kagame. Mass media, mostly via television and radio, also serve to increase community awareness about physical activity and to encourage individuals to become physically active. Car-free free zones created in the cities, exercise grounds, and roads with side walkways for pedestrians represent supportive infrastructure for physical activity. Training of facilitators and establishment of units in charge of physical activity has occurred in most public institutions in Rwanda. Public servants are given 2 hours and related facilitation to participate in physical activity every Friday from 3 p.m. to 5 p.m.

### Interventions Targeting Tobacco Use

Since 17 January 2006, Rwanda has become part of the WHO Framework Convention on Tobacco control and has established various policies targeting tobacco use and, in some cases, also address harmful substances beyond tobacco. Six policies were identified through the online search (Table 2); these include laws, ministerial orders/instructions, and public notices. Instructions of the Minister of Health N° 20/10 of 10/05/2005 (32) intends to protect both non-smokers and the environment from the hazardous effects of public smoking through the promotion of smoke-free spaces and designated smoking spaces, as well as designing visible messages prohibiting smoking in public places. Law N° 08/2013 of 01/03/2013 (33) aims to limit the availability of tobacco, promotes the creation of smoking-free environments, and prohibits the illegal production and trade of tobacco in Rwanda. This law limits the quantity of tobacco for travelers entering Rwanda (this is restricted to 200 cigarettes or cigarillos or 50 cigars or 1 kg of tobacco), and recommend all activities related to tobacco production must be authorized by the Ministry of Trade and Commerce. The law prohibits all tobacco sponsorship and promotion. As part of limiting tobacco accessibility, the law establishing the excise duty (34), which states that tobacco is taxed at 36% of the retail price of a pack of 20 rods plus 130 FRW per pack. Furthermore, Law N°03/2012 of 15/02/2012 (35), regulates the use of narcotic drugs and psychotropic substances and precursors in Rwanda. In addition, the Ministerial order N° 20/33 of 09/06/2015 (36) aims to protect non-smokers and the environment. Places intended for public gatherings such as schools, hospitals, workplaces, pharmacies or sports facilities are designated smoke-free. This order requests that owners of public spaces establish smoking areas with clear warnings against public smoking.

The Ministerial order N° 20/34 of 09/06/2015 (37) regulates the design of health warning messages on tobacco packages. Through this order, all tobacco sold on Rwandan territory is packaged and labeled with clear health warning messages, such as smoking kills, causes cancer, heart diseases, and other health conditions such as impotence, infertility, miscarriages, and stroke. A Ministerial public notice bans water pipe tobacco known as shisha (38) on Rwandan territory.

The stakeholder consultations identified several programs that aim to increase tobacco cessation. Large public awareness campaigns sensitize the community about the health dangers associated with tobacco use. These are delivered through multiple channels, including the media, mass sports campaigns, and community meetings. Medical students and professional associations are involved in campaigns against tobacco use. In secondary schools, anti-tobacco and drug abuse clubs were established. These clubs help to raise awareness of tobacco harm and cessation both at school and in the community.

### Interventions Targeting Harmful Use of Alcohol

No specific policy intervention regulating harmful use of alcohol was found through desk review. However, we identified four cross-cutting policies (Table 2) that target harmful use of alcohol and many more NCDs risk factors in Rwanda. The law establishing the excise duty (34) states that local beer and exported beer are taxed at a rate of 30 and 60%, respectively. Local and imported wines are taxed at a rate of 30 and 70%, respectively while brandies, liquor, and whisky are at a rate of 70%. The stakeholder consultations identified different programs and other preventive interventions targeting the harmful use of alcohol. There are various awareness campaigns to sensitize the community about health hazards associated with the harmful use of alcohol through mass media (radio, television). These same campaigns are also integrated into existing local communication channels, for example through villages meetings, during monthly physical activities campaigns, and after community activities. Through stakeholders' consultations, we identified multi-sectoral operations consisting of different government institutions, namely police, local authorities, regulatory authorities, and staff in charge of health at different levels, jointly identify and ban non-standardized alcohol production and consumption.

As illustrated in Table 3, Rwanda has implemented most of the WHO bet buys recommended interventions targeting tobacco use and physical activity. For the harmful use of alcohol and unhealthy diet, however, there is inadequate implementation of the recommended interventions. For the harmful use of alcohol, these limitations relate to access to retailed alcoholic beverages and alcohol advertisement. For unhealthy diet, there are no interventions to reduce salt intake in food or to promote the replacement of trans-fat food.

**Table 3:** Current implementation of ‘WHO Best Buy' Interventions in Rwanda.

**Risk factors**	**WHO Recommended best buys Interventions**	**Progress**
**Tobacco use**	Tax increases	Fully implemented
	Smoke -free-indoor workplace and public places	Fully implemented
	Health information and warnings against tobacco use	Fully implemented
	Bans on tobacco advertising, promotion and sponsorship	Fully implemented
**Harmful alcohol use**	Tax increases	Fully implemented
	Restricted access to retailed alcohol	Inadequate
	Bans on alcohol advertising	Inadequate
**Unhealthy diet**	Reduced salt intake in food	Inadequate
	Replacement of trans fat with polyunsaturated fat	Inadequate
**Physical activity**	Public awareness campaigns through mass media on physical activity combined motivational and environmental programs aimed at supporting behavioral change of physical activity levels	Fully implemented

Participants representing each of the institutions that participated in this study attended the one-day stakeholders' consultative workshop. The preliminary findings from the situational analysis were well-received and validated. All participants generally agreed with the findings and provided constructive inputs. For example, they all agree on the lack of specific policies targeting harmful use of alcohol.

### Perceived Barriers and Facilitators for the Implementation of Population-Level Interventions Targeting NCD Risk Factors

Ten key informants shared their perspectives and experiences related to implementing population-level interventions. All interviewees confirmed that Rwanda has implemented various interventions targeting risk factors for diabetes and hypertension. They agreed that various obstacles hinder the implementation of different population-level interventions. They openly shared their perception of the barriers as well as what they think can facilitate the implementation of population-level interventions targeting diabetes and hypertension. The findings are classified in twelve themes emerged from the analysis, seven of which represent perceived barriers and five of which represent perceived facilitators. These themes, along with supporting quotes from the key informant interviews, are presented in the following paragraphs.

### Perceived Barriers to the Implementation of Population Level Interventions

#### Limited Knowledge and Misbeliefs About Ongoing Interventions and Targeted Risk Factors

Some of the ongoing prevention activities are misinterpreted among the general population, which may be a reason for low community participation. For instance, physical activity interventions are considered to target only people who have excessively eaten and/or want to lose weight. Similarly, some people still believe that being overweight is a sign of high socio-economic status, instead of being among the risk factors for diabetes and hypertension. These misbeliefs about ongoing interventions and targeted risk factors among the population pose a great barrier to the implementation of population-level interventions.

“*You will meet people in the community who still think that a man with a big tummy is rich and is eating well whereas others think that doing sport is just for overweight people who want to reduce weight”* (KIIP8).

#### Insufficient Mobilization of the Community

Some of the interviewed key informants said that there are plenty of good ongoing population-level interventions targeting risk factors for hypertension and diabetes, but the community is not well mobilized about these interventions to attract their participation, which is a barrier to their successful implementation and scale up to the community level. For example, on physical activity interventions, they (interviewees) urge that using phone short messages to mobilize the community for participation is not enough and does not reach every one especially those who do not own cell phone. They reported a lack of local leader's efforts to mobilize the whole community to get involved in ongoing population-level interventions is a barrier to physical activity participation.

“*Physical activity is a good intervention that can be scaled up at community level countrywide but in addition to the messages spread by different people, have you ever seen local leaders mobilizing those poor people about car-free day to participate? Messages spread by local leaders and community health workers have a big influence on the community therefore they should be involved to mobilize community participation in physical activity”* (KIIP6).

#### Lack of Consistency on the Implementation of Friday Physical Activity Across Institutions

One key informant highlighted issues related to Friday Physical Activity specifically. One issue is that not all public institutions provide sport facility subscription to their employees. Additionally, targeting only the public sector with this intervention may be too restrictive, when many other individuals could potentially also benefit.

“*In some public institution, Friday sport is not done and no facilitation is provided whereas others do, secondly, why this intervention is for public servants only while there is a great number of Rwandans working for private institutions?”* (KIIP10).

#### Limited Collaborative Efforts From All Involved Stakeholders

Targeting risk factors for diabetes and hypertension requires efforts from all relevant stakeholders; however, some key-informants from non-health domains were doubtful of their relevance and insisted that Ministry of health deals with all diseases and related issues. This is indeed a great barrier to implementation of population-level interventions targeting risk factor for hypertension and diabetes since most of these risk factors cross-cut across different sectors. Lack or limited collaborative effort to implement these interventions was identified as a barrier to successful implementation.

“*But I think that is Ministry of Health's mandate, for us, we only deal with food production and their consumption but diseases are dealt by the Ministry of health”* (KIIP3).

#### Conflicts Between Commercial and Public Health Interests

Through various marketing strategies (e.g., betting games, music, shows, and films), industries encourage people to consume beer, tobacco, sugar sweetened beverages and energy drinks. Public health actors, on the other hand, have fewer resources and use routine ways of mobilizing people to live healthy. The influence of this kind of commercial competition is a threat to the implementation of population level intervention targeting alcohol, tobacco and other unhealthy products.

“*You will see many youths attending new beer promotion shows because they have used expensive and attractive ways of marketing; such ways are difficult for us who are protecting them from NCDs. Regarding tobacco, there is also an expensive way of marketing tobacco in the movies using celebrities which then entices people, especially youth, to imitate them,”* (KIIP6).

#### Lack of Adequate Funding for Implementing Population-Level Interventions

Human and financial resources as well as related logistics are required for implementing population-level interventions. Due to a lack of required resources, some interventions are partially implemented. Many interventions are designed to have a countrywide coverage; an interviewed participant reported that they are imposed to implement this in some parts of the country due to resources constraints. Additionally, healthy food is expensive; for example, eating fruits and vegetables is highly recommended for healthy diet, yet they are among the most expensive food types in Rwanda, which makes it difficult to recommend them to the population.

“*It is shameful to tell people to eat fruits when you know that they cannot afford them because of the high cost. The same for some programs; we prefer to implement them in some districts because of financial constraints”* (KIIP4).

#### Limited Policy Enforcement of Population Level Interventions

Lack of follow-up and policy reinforcement was reported as barrier to successful implementation of population-level interventions. Interviewed participants reported that some interventions (policies or programs) are not well implemented due to limited enforcement by implementers, which is perceived as a barrier to their success. For example, public servants are given paid hours for physical activity but some prefer to use this time for different purposes because there is no or limited enforcement.

“*Almost all public servants are given time for physical exercise and are subscribed to the nearest physical exercise facilities but they opt to go for their own businesses during physical activity hours, and those are people who are paid hourly by the government but I have never seen anyone asked to explain why not attending physical activity”* (KIIP5).

Likewise, regardless of the progress to date about tobacco use, some people smoke in public and are not condemned for doing, which shows a weak policy reinforcement. This is a barrier to the protection of non-smokers and the environment in general against tobacco and related hazards.

“*There is an improvement for public smoking reduction but since we do not see people sanctioned for smoking in public; it will still be a problem*,” (KIIP7).

### Perceived Facilitators for the Implementation of Population Level Interventions

#### Paying Special Attention to Higher Risk Groups, Such as the Elderly

Some groups of people, such as the elderly, are more exposed to and burdened by NCDs than others; these population groups, however, are not sufficiently targeted. Having strategies to locate and engage the elderly in ongoing population-level interventions could make a remarkable improvement.

“*Due to their age, old people are vulnerable to most NCDs, they have little strength to go for physical activity, limited medical assistance, they stay at home alone and some of them will tell you that they prefer to die instead of quitting alcohol or tobacco. I truly think this category needs special help. If we are aware of people who are at risk of developing a disease, what do we do to protect them? What do we do to reduce their agony? We do not have these interventions targeting them, for example, there should be a community health worker in charge of NCDs to group old people per village and schedule a regular education session targeting to engage them in population-level interventions. This should be done in an organized way and be reported to make sure they are done progressively”* (KIIP9).

#### Strengthened Community Awareness Campaigns of the Ongoing Interventions

Educating individuals on the risk factors for diabetes and hypertension and how to prevent the diseases will likely enhance compliance and facilitate the implementation of population level interventions. Most participants reported that individuals should be educated about the risk factors from early childhood to mitigate misconceptions. Interviewed participants highlighted specific modalities, such the use of multiple strategies for community awareness campaigns such as through regular and locally known dramas or church leaders, which could improve the impact of health messaging in the community.

“*It is about educating the population regarding the interventions and when they understand their roles and responsibility; they will surely comply and this will lead to an increased impact. Church leaders, schools, and radio-based famous drama like urunana and musekeweya (Kinyarwanda named dramas) are good communication channels to reach many people”* (KIIP9).

#### Strengthened Multi-Sectoral Collaboration and Strengthen Stakeholders' Involvement in Designing and Planning Population-Level Interventions

Risk factors for diabetes and hypertension cut across multiple sectors and cannot be managed by health sector alone. Participants emphasized the importance of engaging these multiple stakeholders in designing, planning and implementing population-level interventions. Each stakeholder (ministry and government agencies as well as NGOs and other private sector institutions should have play their specific role in NCDs prevention.

“*Each of the related ministries and institutions has a role in preventing NCDs; for example, laws prohibiting public smoking: Rwanda revenue authority intervenes by increasing tobacco-related products taxes and ministry of health by providing coordination. Ministry of education in partnership with Ministry of health can help in strengthening NCD prevention teaching from primary to the secondary schools”* (KIIP4).

#### Increased Funds Allocated to Population-Level

Most of the interviewed participants reported limited resources allocated to population level interventions. Implementing agencies should have sufficient budget allocated to awareness campaigns in the community and different NCDs screening. Government should be attentive to NCDs burden and avail sufficient budget to run different ongoing population-level intervention targeting NCDs risk factors.

“*I don't know how much the government allocates in the health sector, but prevention of non-communicable diseases requires sufficient resources to reach the whole community”* (KIIP2).

#### Reinforced Policy Interventions Targeting Tobacco Use and Harmful Use of Alcohol

There are number of laws regulating production, importation and distribution of tobacco and alcohol. Interviewed participants insisted that such laws should be reinforced in order to better control concerned risk factors. For example, a participant suggested that any kind of advertisement for alcohol, tobacco and any risky products should be abolished to facilitate their control; otherwise, it is difficult to convince the community about their harmfulness of them when they being publically advertised in the country.

“*You cannot convince people that alcohol is dangerous when it is being advertised in different ways, laws against alcohol advertising should be established and applied”* (KIIP6).

## Discussion

In response to the increasing burden of NCDs, this study identified and described population-level interventions in Rwanda targeting risk factors for diabetes and hypertension, as well as perceived barriers and facilitators for the implementation of these interventions. The NCD policy was identified as the main policy targeting risk factors for diabetes and hypertension, and other identified policies included those related to food and nutrition, physical activity promotion, and tobacco control. Most WHO best buy interventions targeting tobacco use and physical activity have been adopted, while those targeting harmful use of alcohol and unhealthy diet were inadequately implemented.

This study identified multi-layered and multiple-component interventions targeting unhealthy diet. The promotion of village and household gardens for the growing of vegetables and fruits was one such intervention identified. These interventions were primarily initiated targeting malnutrition than actually NCD, for example the kitchen garden aims to combat malnutrition among children but can indirectly target unhealthy diet as NCDs risk factor. Studies have revealed that the promotion of vegetable and fruits production has been effective in reducing population exposure to unhealthy diets (39) and agricultural policies increase healthy food production and accessibility (40), and reduce its cost at the market (41). Nutrition education in schools has been effective in promoting students' healthy diet habits in other studies (42). Salt reduction is a key intervention targeting unhealthy diet and one of the WHO best buy interventions targeting (43), however, no related interventions were identified in this study. This was similarly reported in the recent NCDs Diseases Progress Monitor 2022 (44) where implementation of best buys recommended intervention targeting unhealthy diet were partial or inadequate. As country progresses to ensure reduction of poverty and malnutrition, unhealthy diet as a risk factor need to be particularly considered.

Regarding physical inactivity (PA), different campaigns identified in Rwanda focused on mass media, school sport competitions, mass sports campaigns, and the construction of physical activity infrastructures in the community. This study found strong government willingness to set up policies and a supporting environment to encourage physical activity through different initiatives, such as community mass sports campaigns known as car-free day, Friday physical activity for public servants, and different inter-institutional as well as school sport competitions. Similar interventions have worked worldwide (45) and are in line with WHO best buys recommended interventions targeting physical inactivity (43).

Concerning interventions targeting tobacco smoking in Rwanda, we identified interventions aimed at raising community awareness of the dangers of tobacco use, restricted access to tobacco and creating smoke-free areas. These interventions are all in line with WHO best buys recommendations for tobacco control. This study observed a political willingness to address tobacco as a risk factor, through the formulation of tobacco control laws, civil society involvement in anti-tobacco campaigns, and public education about tobacco use-related health risks. These findings support what is reported broadly; for example, It has been shown that involving different sectors in implementation of interventions targeting tobacco use is an important factor for their success (46), and that education combined with other measures such as excise taxes and prices on tobacco products help to encourage smoking cessation (47). This study identified no specific policies targeting the harmful use of alcohol but identified cross-cutting policies with relevant components. Such components include, for example, awareness campaigns emphasizing the health risks of harmful use of alcohol, and banning non-standardized alcohol production. A lack of sufficiently coordinated policies targeting the harmful use of alcohol will likely allow alcohol industries to promote, market, and lobby their products (48, 49).

All identified population-level interventions in this study were not evaluated. An evaluation is essential part of interventions to monitor the intended objectives and for a better planning. Through some of these interventions were newly introduced, there other which would have been evaluated. Most of the interventions target all age categories of the population with countrywide coverage. This ensure that every one can benefits from these interventions, however, few interventions such Friday physical activity intervention only target subset of the population, this can be scaled up to benefits more people. We identified different barriers perceived to be hindering the implementation of some population-level interventions.

Inconsistency in implementing physical activity interventions across different settings was identified as a barrier. A scarcity of resources, the nature of work for policymakers, who are juggling countless responsibilities and priorities, and the lack of exposure to the relevant burden may be linked to this inconsistency. People in cities, for example, are on average more exposed to sedentary life than those in rural areas (17); this may explain why some interventions, such as those promoting physical activity, are more prevalent in urban. However, successful interventions are being scaled countrywide. Car-free day, for example, started in Kigali city and is currently being scaled up to promote physical activity across much of the country.

Limited policy enforcement was identified as a barrier, especially linked to tobacco use, similar to a study assessing tobacco control in 17 HICs and LMICs that ratified the Framework Convention on Tobacco Control (FCTC), which found a weak implementation of tobacco control policies, particularly in developing countries (50). Research conducted in 16 countries found significantly more tobacco advertisements and outlets in LMICs compared with HICs (51). Tobacco smoking is still common throughout entertainment (music and video clips) in Rwanda. Commercial interests of tobacco industries negatively affect tobacco control policies (52). Overall, limited knowledge and misbeliefs about ongoing interventions and targeted risk factors was reported as a barrier to implementation of various population-level interventions. This barrier may be linked to central planning and low community involvement in designing and planning of these interventions. Insufficient mobilization of the community was reported as a barrier to implementing population-level interventions, this indeed lead to low involvement and buy in by the community members. In Rwanda, due to scarcity of medical and public health professional (53), there might be not enough people equipped with sufficient knowledge about available to mobilize the community about ongoing prevention interventions targeting diabetes and hypertension. Limited knowledge and misbelief may lead to low buy-in by the community and hinder the effectiveness of the concerned intervention. The literature recommends cooperating with the population when designing population-level interventions to ensure culturally adapted and understandable interventions (54).

Weak collaboration among various stakeholders was identified as a barrier to the implementation of population-level intervention targeting risk factors for diabetes and hypertension in Rwanda. This finding was similarly found in various settings such as in Kenya, Malawi, and Mauritius (55, 56).

Lack of NCD units or focal persons in non-health sector institutions might lead to this weakness. Coordination of all involved sectors to monitor individual stakeholders' roles and contributions toward preventing various risk factors would likely lead to improved control of NCDs at the population-level.

Limited funding was also a barrier to the implementation of population-level intervention targeting risk factors for diabetes and hypertension. This likely plays a role in the inconsistent and partial implementation of some interventions. According to Rwanda NCDs strategic plan (2014–2019), NCD prevention in Rwanda is generally underfunded (26) and was commonly reported in other settings (57, 58).

This study found a number of perceived facilitators which could be considered to improve for population-level interventions implementation. Special attention paid to interventions explicitly designed to protect the elderly, as they experience the highest burden due to NCDs. Thus focusing on high-risk population groups, such as the elderly, is likely a facilitator for effectively addressing NCDs in Rwanda. Interventions to improve physical activity (59) and home-based strategies encouraging fruits and vegetable consumptions (60) specifically among the elderly have been implemented successfully, for example in South Africa and the United States, and could be model for adaption in Rwanda or elsewhere. Strengthened community awareness campaigns and strengthened multi-sectoral collaboration and strengthen stakeholders' involvement in designing and planning of the ongoing interventions is essential and would be of a great importance to rise community and stakeholders knowledge and engagement in on going intervention. It is indeed reported as a key to successful implementation of NCDs prevention interventions in other settings (61, 62). Training and involving more community members in design and implementation of these intervention would be a locally made solution to limited health professionals involved in these activities. Since NCDs risk factors cross cut across different sectors, strengthened multi-sectoral collaboration is widely recommended to facilitate the implementation of population-level interventions targeting NCDs (63). Increased funds allocated to population-level was reported a facilitator for implementing population-level interventions, various studies revealed the role of funding in NCDs preventions (64, 65). The budget allocated for health sector has been revised upward over the past 5 years (66), which shows the government willingness to respond to health issues in the country including the most emerging NCDs. Reinforcement of policy interventions targeting tobacco use and harmful use of alcohol was perceived as a key facilitator to their implementation. Few interventions targeting harmful use of alcohol were identified in this study, which shows a need for reinforcement. The same for tobacco, public smoking was reported to be practiced with no rigorous prevention measures. Filling the gaps between policy and their implementation is the ideal way to successfully prevent NCDs risk factors (67).

This situational analysis comprised a desk review, which included an online search as well as consultations with stakeholders, key informant interviews, and a consultative workshop. We defined the methods a priori and conducted each step with methodological rigor; however, there were some study limitations; searches only focused on a single governmental website to locate policy documents. It is possible, that other documents exist that we did not identify. However, we supplemented these searches with input from stakeholders, who are also familiar with the policy landscape in Rwanda. Regarding the key informant interviews, only 10 key informants were interviewed; in selecting these key informants, however, we aimed to ensure that multiple and differing perspectives important for the design and implementation of population-level NCD interventions would be captured. Talking to the community as direct beneficiaries for the population-level interventions would possibly identify further barriers and facilitator beyond implementers' perspectives.

## Conclusion

This situational analysis of current population-level interventions, as well as perceived barriers and facilitators affecting the implementation. Various interventions are implemented, their evaluation is needed to monitor the effectiveness. While barriers and facilitators have been reported, these findings provide useful input to inform the strengthening of the public health response to diabetes and hypertension in Rwanda.

## Data Availability Statement

The raw data supporting the conclusions of this article will be made available by the authors, without undue reservation.

## Ethics Statement

The studies involving human participants were reviewed and approved by the Rwanda National Ethics Committee Approval No. 0025/RNEC/2019. The participants provided their written informed consent to participate in this study.

## Author Contributions

JPN and DT were involved in protocol development, data collection, analysis, conception, and successive revision of the manuscript. SN, JBN, and GU were involved in protocol development, data collection and analysis, reviewed, and added inputs in the manuscript. CB, JCB, SR, and TY were involved in protocol development, reviewed, and added inputs in the manuscript. ER and JB were involved in protocol development (methodology), reviewed, and added inputs in the manuscript. All authors contributed to the article and approved the submitted version.

## Funding

This work was supported by the German Federal Ministry of Education and Research [Bundesministerium für Bildung und Forschung (BMBF)] (01KA1608) as part of the Research Networks for Health Innovation in Sub-Saharan Africa funding initiative. The funder had no role in designing the study or in writing the manuscript.

## Conflict of Interest

The authors declare that the research was conducted in the absence of any commercial or financial relationships that could be construed as a potential conflict of interest.

## Publisher's Note

All claims expressed in this article are solely those of the authors and do not necessarily represent those of their affiliated organizations, or those of the publisher, the editors and the reviewers. Any product that may be evaluated in this article, or claim that may be made by its manufacturer, is not guaranteed or endorsed by the publisher.
